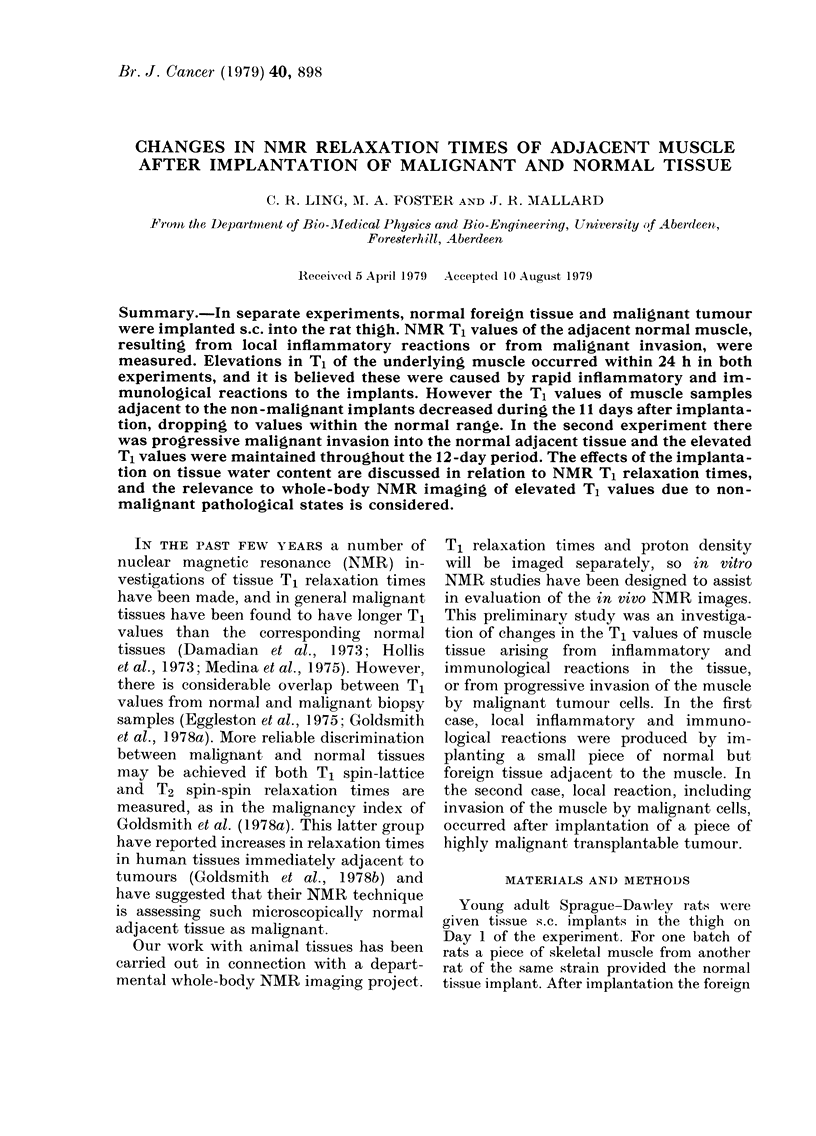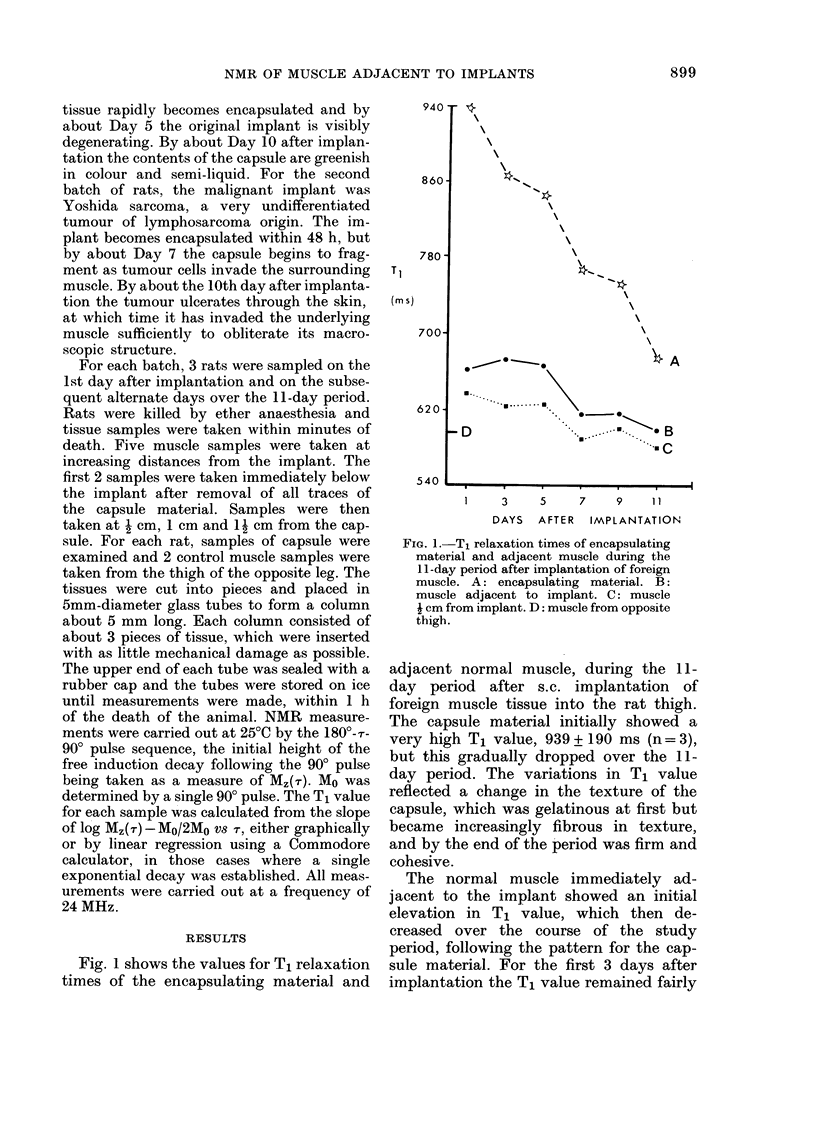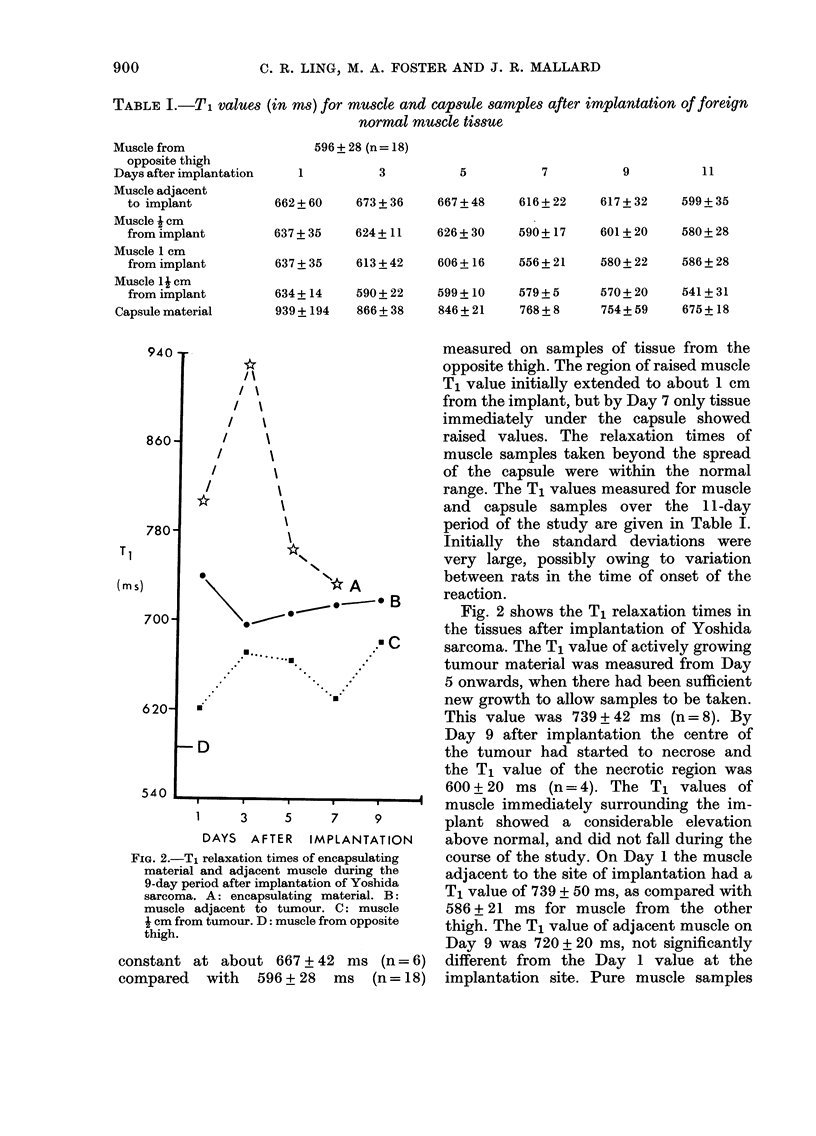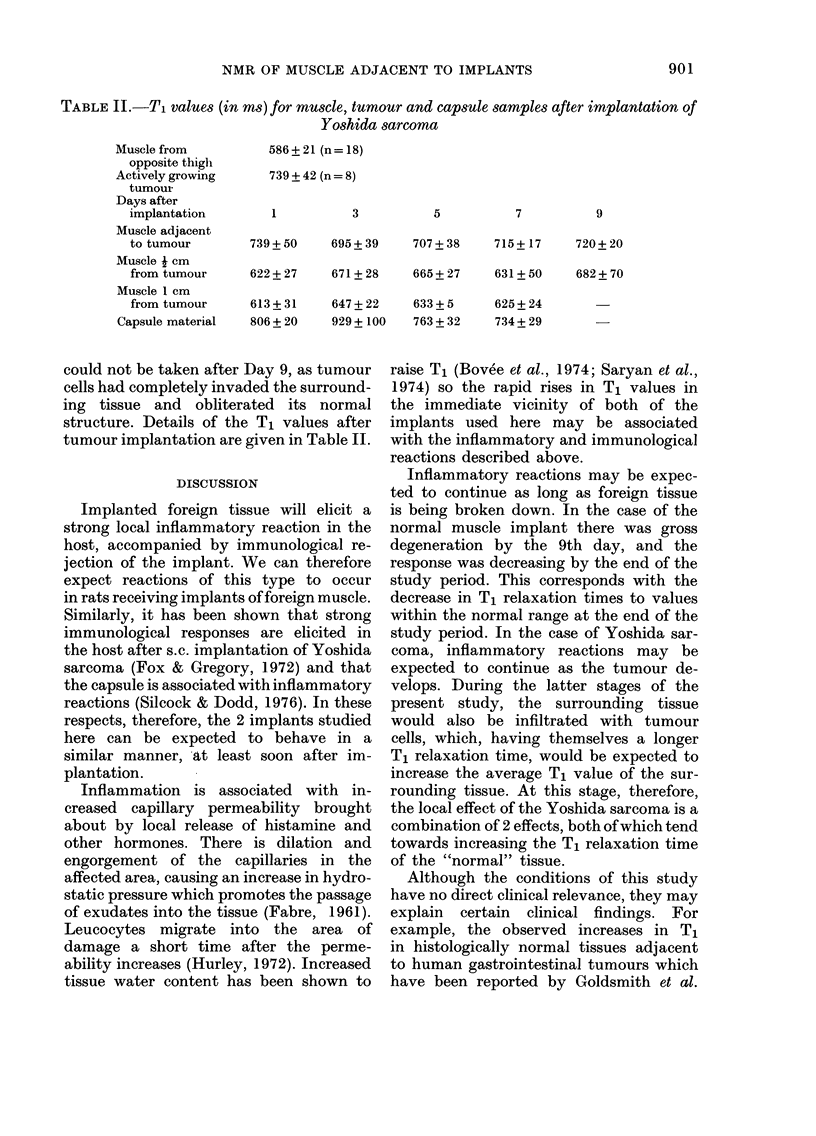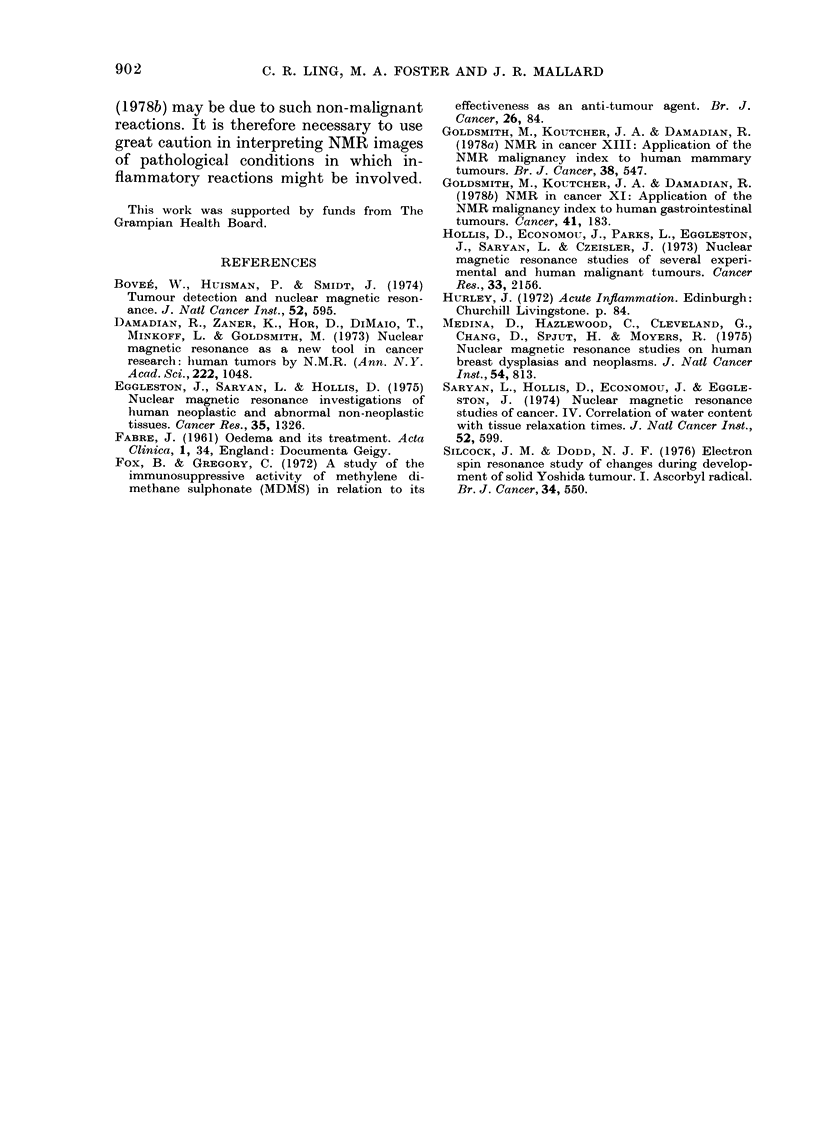# Changes in NMR relaxation times of adjacent muscle after implantation of malignant and normal tissue.

**DOI:** 10.1038/bjc.1979.283

**Published:** 1979-12

**Authors:** C. R. Ling, M. A. Foster, J. R. Mallard

## Abstract

In separate experiments, normal foreign tissue and malignant tumour were implanted s.c. into the rat thigh. NMR T1 values of the adjacent normal muscle, resulting from local inflammatory reactions or from malignant invasion, were measured. Elevations in T1 of the underlying muscle occurred within 24 h in both experiments, and it is believed these were caused by rapid inflammatory and immunological reactions to the implants. However the T1 values of muscle samples adjacent to the non-malignant implants decreased during the 11 days after implantation, dropping to values within the normal range. In the second experiment there was progressive malignant invasion into the normal adjacent tissue and the elevated T1 values were maintained throughout the 12-day period. The effects of the implantation on tissue water content are discussed in relation to NMR T1 relaxation times, and the relevance to whole-body NMR imaging of elevated T1 values due to nonmalignant pathological states is considered.


*Br. J. Cancer* (1979) **40**, 898

### CHANGES IN NMR RELAXATION TIMES OF ADJACENT MUSCLE AFTER IMPLANTATION OF MALIGNANT AND NORMAL TISSUE

C. R. LING, M. A. FOSTER AND J. R. MALLARD

*From the Department of Bio-Medical Physics and Bio-Engineering, University of Aberdeen, Foresterhill, Aberdeen*

Received 5 April 1979 Accepted 10 August 1979

**Summary.—In separate experiments, normal foreign tissue and malignant tumour were implanted s.c. into the rat thigh. NMR $T_1$ values of the adjacent normal muscle, resulting from local inflammatory reactions or from malignant invasion, were measured. Elevations in $T_1$ of the underlying muscle occurred within 24 h in both experiments, and it is believed these were caused by rapid inflammatory and immunological reactions to the implants. However the $T_1$ values of muscle samples adjacent to the non-malignant implants decreased during the 11 days after implantation, dropping to values within the normal range. In the second experiment there was progressive malignant invasion into the normal adjacent tissue and the elevated $T_1$ values were maintained throughout the 12-day period. The effects of the implantation on tissue water content are discussed in relation to NMR $T_1$ relaxation times, and the relevance to whole-body NMR imaging of elevated $T_1$ values due to non-malignant pathological states is considered.**

IN THE PAST FEW YEARS a number of nuclear magnetic resonance (NMR) investigations of tissue $T_1$ relaxation times have been made, and in general malignant tissues have been found to have longer $T_1$ values than the corresponding normal tissues (Damadian *et al.*, 1973; Hollis *et al.*, 1973; Medina *et al.*, 1975). However, there is considerable overlap between $T_1$ values from normal and malignant biopsy samples (Eggleston *et al.*, 1975; Goldsmith *et al.*, 1978*a*). More reliable discrimination between malignant and normal tissues may be achieved if both $T_1$ spin-lattice and $T_2$ spin-spin relaxation times are measured, as in the malignancy index of Goldsmith *et al.* (1978*a*). This latter group have reported increases in relaxation times in human tissues immediately adjacent to tumours (Goldsmith *et al.*, 1978*b*) and have suggested that their NMR technique is assessing such microscopically normal adjacent tissue as malignant.

Our work with animal tissues has been carried out in connection with a departmental whole-body NMR imaging project. $T_1$ relaxation times and proton density will be imaged separately, so *in vitro* NMR studies have been designed to assist in evaluation of the *in vivo* NMR images. This preliminary study was an investigation of changes in the $T_1$ values of muscle tissue arising from inflammatory and immunological reactions in the tissue, or from progressive invasion of the muscle by malignant tumour cells. In the first case, local inflammatory and immunological reactions were produced by implanting a small piece of normal but foreign tissue adjacent to the muscle. In the second case, local reaction, including invasion of the muscle by malignant cells, occurred after implantation of a piece of highly malignant transplantable tumour.

### MATERIALS AND METHODS

Young adult Sprague–Dawley rats were given tissue s.c. implants in the thigh on Day 1 of the experiment. For one batch of rats a piece of skeletal muscle from another rat of the same strain provided the normal tissue implant. After implantation the foreign tissue rapidly becomes encapsulated and by about Day 5 the original implant is visibly degenerating. By about Day 10 after implantation the contents of the capsule are greenish in colour and semi-liquid. For the second batch of rats, the malignant implant was Yoshida sarcoma, a very undifferentiated tumour of lymphosarcoma origin. The implant becomes encapsulated within 48 h, but by about Day 7 the capsule begins to fragment as tumour cells invade the surrounding muscle. By about the 10th day after implantation the tumour ulcerates through the skin, at which time it has invaded the underlying muscle sufficiently to obliterate its macroscopic structure.

For each batch, 3 rats were sampled on the 1st day after implantation and on the subsequent alternate days over the 11-day period. Rats were killed by ether anaesthesia and tissue samples were taken within minutes of death. Five muscle samples were taken at increasing distances from the implant. The first 2 samples were taken immediately below the implant after removal of all traces of the capsule material. Samples were then taken at ½ cm, 1 cm and 1½ cm from the capsule. For each rat, samples of capsule were examined and 2 control muscle samples were taken from the thigh of the opposite leg. The tissues were cut into pieces and placed in 5mm-diameter glass tubes to form a column about 5 mm long. Each column consisted of about 3 pieces of tissue, which were inserted with as little mechanical damage as possible. The upper end of each tube was sealed with a rubber cap and the tubes were stored on ice until measurements were made, within 1 h of the death of the animal. NMR measurements were carried out at 25°C by the 180°-$\tau$-90° pulse sequence, the initial height of the free induction decay following the 90° pulse being taken as a measure of $M_z(\tau)$. $M_0$ was determined by a single 90° pulse. The $T_1$ value for each sample was calculated from the slope of log $M_z(\tau) - M_0/2M_0$ *vs* $\tau$, either graphically or by linear regression using a Commodore calculator, in those cases where a single exponential decay was established. All measurements were carried out at a frequency of 24 MHz.

### RESULTS

Fig. 1 shows the values for $T_1$ relaxation times of the encapsulating material and

FIG. 1.—$T_1$ relaxation times of encapsulating material and adjacent muscle during the 11-day period after implantation of foreign muscle. A: encapsulating material. B: muscle adjacent to implant. C: muscle ½ cm from implant. D: muscle from opposite thigh.

adjacent normal muscle, during the 11-day period after s.c. implantation of foreign muscle tissue into the rat thigh. The capsule material initially showed a very high $T_1$ value, $939 \pm 190$ ms ($n = 3$), but this gradually dropped over the 11-day period. The variations in $T_1$ value reflected a change in the texture of the capsule, which was gelatinous at first but became increasingly fibrous in texture, and by the end of the period was firm and cohesive.

The normal muscle immediately adjacent to the implant showed an initial elevation in $T_1$ value, which then decreased over the course of the study period, following the pattern for the capsule material. For the first 3 days after implantation the $T_1$ value remained fairly

TABLE I.—*$T_1$ values (in ms) for muscle and capsule samples after implantation of foreign normal muscle tissue*

| Muscle from opposite thigh | 596 ± 28 (n = 18) | | | | | |
|---|---|---|---|---|---|---|
| Days after implantation | 1 | 3 | 5 | 7 | 9 | 11 |
| Muscle adjacent to implant | 662 ± 60 | 673 ± 36 | 667 ± 48 | 616 ± 22 | 617 ± 32 | 599 ± 35 |
| Muscle ½ cm from implant | 637 ± 35 | 624 ± 11 | 626 ± 30 | 590 ± 17 | 601 ± 20 | 580 ± 28 |
| Muscle 1 cm from implant | 637 ± 35 | 613 ± 42 | 606 ± 16 | 556 ± 21 | 580 ± 22 | 586 ± 28 |
| Muscle 1½ cm from implant | 634 ± 14 | 590 ± 22 | 599 ± 10 | 579 ± 5 | 570 ± 20 | 541 ± 31 |
| Capsule material | 939 ± 194 | 866 ± 38 | 846 ± 21 | 768 ± 8 | 754 ± 59 | 675 ± 18 |

FIG. 2.—$T_1$ relaxation times of encapsulating material and adjacent muscle during the 9-day period after implantation of Yoshida sarcoma. A: encapsulating material. B: muscle adjacent to tumour. C: muscle ½ cm from tumour. D: muscle from opposite thigh.

constant at about 667 ± 42 ms (n = 6) compared with 596 ± 28 ms (n = 18) measured on samples of tissue from the opposite thigh. The region of raised muscle $T_1$ value initially extended to about 1 cm from the implant, but by Day 7 only tissue immediately under the capsule showed raised values. The relaxation times of muscle samples taken beyond the spread of the capsule were within the normal range. The $T_1$ values measured for muscle and capsule samples over the 11-day period of the study are given in Table I. Initially the standard deviations were very large, possibly owing to variation between rats in the time of onset of the reaction.

Fig. 2 shows the $T_1$ relaxation times in the tissues after implantation of Yoshida sarcoma. The $T_1$ value of actively growing tumour material was measured from Day 5 onwards, when there had been sufficient new growth to allow samples to be taken. This value was 739 ± 42 ms (n = 8). By Day 9 after implantation the centre of the tumour had started to necrose and the $T_1$ value of the necrotic region was 600 ± 20 ms (n = 4). The $T_1$ values of muscle immediately surrounding the implant showed a considerable elevation above normal, and did not fall during the course of the study. On Day 1 the muscle adjacent to the site of implantation had a $T_1$ value of 739 ± 50 ms, as compared with 586 ± 21 ms for muscle from the other thigh. The $T_1$ value of adjacent muscle on Day 9 was 720 ± 20 ms, not significantly different from the Day 1 value at the implantation site. Pure muscle samples

TABLE II.—*$T_1$ values (in ms) for muscle, tumour and capsule samples after implantation of Yoshida sarcoma*

| | | | | | |
|---|---|---|---|---|---|
| Muscle from opposite thigh | 586 ± 21 (n = 18) | | | | |
| Actively growing tumour | 739 ± 42 (n = 8) | | | | |
| Days after implantation | 1 | 3 | 5 | 7 | 9 |
| Muscle adjacent to tumour | 739 ± 50 | 695 ± 39 | 707 ± 38 | 715 ± 17 | 720 ± 20 |
| Muscle ½ cm from tumour | 622 ± 27 | 671 ± 28 | 665 ± 27 | 631 ± 50 | 682 ± 70 |
| Muscle 1 cm from tumour | 613 ± 31 | 647 ± 22 | 633 ± 5 | 625 ± 24 | — |
| Capsule material | 806 ± 20 | 929 ± 100 | 763 ± 32 | 734 ± 29 | — |

could not be taken after Day 9, as tumour cells had completely invaded the surrounding tissue and obliterated its normal structure. Details of the $T_1$ values after tumour implantation are given in Table II.

### DISCUSSION

Implanted foreign tissue will elicit a strong local inflammatory reaction in the host, accompanied by immunological rejection of the implant. We can therefore expect reactions of this type to occur in rats receiving implants of foreign muscle. Similarly, it has been shown that strong immunological responses are elicited in the host after s.c. implantation of Yoshida sarcoma (Fox & Gregory, 1972) and that the capsule is associated with inflammatory reactions (Silcock & Dodd, 1976). In these respects, therefore, the 2 implants studied here can be expected to behave in a similar manner, at least soon after implantation.

Inflammation is associated with increased capillary permeability brought about by local release of histamine and other hormones. There is dilation and engorgement of the capillaries in the affected area, causing an increase in hydrostatic pressure which promotes the passage of exudates into the tissue (Fabre, 1961). Leucocytes migrate into the area of damage a short time after the permeability increases (Hurley, 1972). Increased tissue water content has been shown to raise $T_1$ (Bovée *et al.*, 1974; Saryan *et al.*, 1974) so the rapid rises in $T_1$ values in the immediate vicinity of both of the implants used here may be associated with the inflammatory and immunological reactions described above.

Inflammatory reactions may be expected to continue as long as foreign tissue is being broken down. In the case of the normal muscle implant there was gross degeneration by the 9th day, and the response was decreasing by the end of the study period. This corresponds with the decrease in $T_1$ relaxation times to values within the normal range at the end of the study period. In the case of Yoshida sarcoma, inflammatory reactions may be expected to continue as the tumour develops. During the latter stages of the present study, the surrounding tissue would also be infiltrated with tumour cells, which, having themselves a longer $T_1$ relaxation time, would be expected to increase the average $T_1$ value of the surrounding tissue. At this stage, therefore, the local effect of the Yoshida sarcoma is a combination of 2 effects, both of which tend towards increasing the $T_1$ relaxation time of the "normal" tissue.

Although the conditions of this study have no direct clinical relevance, they may explain certain clinical findings. For example, the observed increases in $T_1$ in histologically normal tissues adjacent to human gastrointestinal tumours which have been reported by Goldsmith *et al.*

(1978*b*) may be due to such non-malignant reactions. It is therefore necessary to use great caution in interpreting NMR images of pathological conditions in which inflammatory reactions might be involved.

This work was supported by funds from The Grampian Health Board.

REFERENCES

BOVEÉ, W., HUISMAN, P. & SMIDT, J. (1974) Tumour detection and nuclear magnetic resonance. *J. Natl Cancer Inst.*, **52**, 595.

DAMADIAN, R., ZANER, K., HOR, D., DIMAIO, T., MINKOFF, L. & GOLDSMITH, M. (1973) Nuclear magnetic resonance as a new tool in cancer research: human tumors by N.M.R. (*Ann. N.Y. Acad. Sci.*, **222**, 1048.

EGGLESTON, J., SARYAN, L. & HOLLIS, D. (1975) Nuclear magnetic resonance investigations of human neoplastic and abnormal non-neoplastic tissues. *Cancer Res.*, **35**, 1326.

FABRE, J. (1961) Oedema and its treatment. *Acta Clinica*, **1**, 34, England: Documenta Geigy.

FOX, B. & GREGORY, C. (1972) A study of the immunosuppressive activity of methylene dimethane sulphonate (MDMS) in relation to its effectiveness as an anti-tumour agent. *Br. J. Cancer*, **26**, 84.

GOLDSMITH, M., KOUTCHER, J. A. & DAMADIAN, R. (1978*a*) NMR in cancer XIII: Application of the NMR malignancy index to human mammary tumours. *Br. J. Cancer*, **38**, 547.

GOLDSMITH, M., KOUTCHER, J. A. & DAMADIAN, R. (1978*b*) NMR in cancer XI: Application of the NMR malignancy index to human gastrointestinal tumours. *Cancer*, **41**, 183.

HOLLIS, D., ECONOMOU, J., PARKS, L., EGGLESTON, J., SARYAN, L. & CZEISLER, J. (1973) Nuclear magnetic resonance studies of several experimental and human malignant tumours. *Cancer Res.*, **33**, 2156.

HURLEY, J. (1972) *Acute Inflammation*. Edinburgh: Churchill Livingstone. p. 84.

MEDINA, D., HAZLEWOOD, C., CLEVELAND, G., CHANG, D., SPJUT, H. & MOYERS, R. (1975) Nuclear magnetic resonance studies on human breast dysplasias and neoplasms. *J. Natl Cancer Inst.*, **54**, 813.

SARYAN, L., HOLLIS, D., ECONOMOU, J. & EGGLESTON, J. (1974) Nuclear magnetic resonance studies of cancer. IV. Correlation of water content with tissue relaxation times. *J. Natl Cancer Inst.*, **52**, 599.

SILCOCK, J. M. & DODD, N. J. F. (1976) Electron spin resonance study of changes during development of solid Yoshida tumour. I. Ascorbyl radical. *Br. J. Cancer*, **34**, 550.